# A Frequent Mutation in the *FTL* Gene Causing Hyperferritinemia Cataract Syndrome in Turkish Population Is c.-160A>G

**DOI:** 10.4274/tjh.galenos.2018.2018.0194

**Published:** 2019-02-07

**Authors:** Burhan Balta, Murat Erdoğan, Aslıhan Kiraz, Serdal Korkmaz, Alperen Ağadayı

**Affiliations:** 1 Kayseri Training and Research Hospital, Clinic of Medical Genetics, Kayseri, Turkey; 2 Kayseri Training and Research Hospital, Clinic of Hematology, Kayseri, Turkey; 3 Kayseri Training and Research Hospital, Clinic of Ophthalmology, Kayseri, Turkey

**Keywords:** Hyperferritinemia cataract syndrome, FTL, Ferritin, Cataract, Hyperferritinemia

## Abstract

**Objective:**

Hyperferritinemia cataract syndrome (HFCS) is an autosomal dominantly inherited disease characterized by increased serum ferritin levels and bilateral cataract formation in the early period of life. Heterozygote mutations in the 5’ untranslated region of the L-ferritin gene (*FTL*) have been reported to cause this disease. In this study, our purpose was to research the *FTL* gene mutations that cause HFCS in Central Anatolia and the clinical effects of these mutations.

**Materials and Methods:**

Seventeen patients from 6 families with high ferritin levels in performed serum measurements, those who were found to have cataracts in eye examinations, and families with vertical inheritance, since the disease is autosomal dominant, were included in the study. Exons, exon-intron boundaries, and 5’ and 3’ untranslated regions of *FTL* (NM_000146) were sequenced using the Sanger sequencing method.

**Results:**

The female/male ratio of the patients was 7/10. All of the patients were found to have c.-160A>G heterozygous mutation in the *FT*L gene.

**Conclusion:**

In the Turkish population, the prevalence of HFCS is about 1/100,000 and the commonly observed mutation is c.-160A>G mutation.

## Abstract (tr)

**Amaç:**

Hiperferritinemi katarakt sendromu (HFKS) serum ferritin seviyelerinde artış, hayatın erken döneminde bilateral katarakt oluşumu ile karakterize otozomal dominant geçişli genetik bir hastalıktır. L-ferritin geninin (*FTL*) 5’ kodlanmayan bölgesindeki heterozigot mutasyonların bu hastalığa neden olduğu bildirilmiştir. Biz bu çalışmada Orta Anadolu’da HFKS’ye neden olan *FTL* gen mutasyonlarını ve klinik etkilerini araştırmayı amaçladık.

**Gereç ve Yöntemler:**

Serum ölçümünde ferritin yüksekliği olan, göz muayenesinde kataraktı çıkan, hastalık otozomal dominant olduğu için dikey kalıtım gösteren 6 aileden 17 hasta çalışmaya dahil edildi. *FT*L geninin (NM_000146) ekzon, ekzon-intron bağlantı noktaları ve 5’ ve 3’ kodlamayan bölgeleri sanger dizileme yöntemi kullanılarak dizilendi.

**Bulgular:**

Hastalardaki kadın/erkek oranı 7/10 idi. Tüm hastalarda *FTL* geninde c.-160A>G heterozigot mutasyonu tespit edilmiştir.

**Sonuç:**

Türk popülasyonunda HFKS prevelansı yaklaşık 1/100.000’dir ve yaygın görülen mutasyon c.-160A>G’dir.

## Introduction

Cataract is defined as the opacification of the lens of the eye resulting in a decrease in vision. About 30% of congenital cataracts are due to monogenic reasons and they show autosomal dominant inheritance. To date, about 25 related genes have been defined [1]. Hyperferritinemia cataract syndrome (HFCS) (OMIM 600886) is an autosomal dominantly inherited disease characterized by increased serum ferritin levels and bilateral cataract formation in the early period of life. It was first defined by Girelli et al. [2] and Bonneau et al. [3] and independently of each other in 1995. Heterozygote mutations in the 5’ untranslated region of the L-ferritin gene (*FTL*) have been reported to cause this disease [4]. To date, 37 mutations have been defined in the *FTL* gene [5]. There are no literature data about its prevalence in the Turkish population. However, it is estimated to be about 1:200,000 in Australia [6]. Although one case was reported from Turkey in the literature [7], there are no published research studies regarding which mutations are frequent or the clinical effects of these mutations. In this study, our purpose was to research the *FTL* gene mutations that cause HFCS in Central Anatolia and the clinical effects of these mutations.

## Materials and Methods

### Study Group

Seventeen patients from 6 families who were referred to the Kayseri Training and Research Hospital’s Medical Genetics Department from the Hematology and Ophthalmology Department with a prediagnosis of HFCS between June 2014 and December 2017 were included in the study. The study was carried out in the Kayseri Training and Research Hospital’s Medical Genetics Department. The study was approved by the Local Ethics Research Committee of Erciyes University with protocol number 2018/186 and conducted in accordance with the Declaration of Helsinki and good clinical practice guidelines. All subjects or their legal guardians provided written informed consent prior to participation in the study.

Families with high ferritin levels in serum measurements, those who were found to have cataracts in eye examinations, and families with vertical inheritance, as the disease is autosomal dominant, were included in the study. Families with other diseases that cause high ferritin levels, such as hemochromatosis, were excluded from the study.

### Sanger Sequencing Analysis

Genomic DNA was extracted from peripheral blood samples using a DNA isolation kit according to the manufacturer’s instructions (Zinexts Life Science Corp., Taiwan). Exons, exon-intron boundaries, and 5’ and 3’ untranslated regions of *FTL* (NM_000146) were sequenced using appropriate primers by using the Sanger sequence method. PCR conditions were as follows: initial denaturation at 94 °C for 5 min; 35 cycles at 94 °C for 30 s and 58 °C for 45 s; 72 °C for 1 min; and a final extension at 72 °C for 5 min. The PCR products were observed with 2% agarose gel electrophoresis. PCR products with enzyme transition were purified using the Exo-SAP kit (Exo-SAP PCR purification kit, UAB Corporation, USA). The cycle sequence was amplified using BigDye Terminator (Thermo Fisher Scientific, USA), and extension products were purified using Sephadex. The product was sequenced in both strands initiating from the forward and the reverse primers used in the initial PCR and analyzed on an ABI 3500 Genetic Analyzer (Applied Biosystems, Hitachi, Japan). Bioinformatic analysis was conducted using the SeqScape v2.6 program.

## Results

Seventeen patients from 6 families were evaluated. The female/male ratio of the patients was 7/10. The youngest patient was 1 year old, while the oldest patient was 72 years old and the average age was 33.3±19.5. Patients F1P3, F3P7, F3P8, F3P9, F5P12, and F5P16 had not undergone cataract operation yet. The patients’ cataract operation ages also differed. Patient F2P5 had been operated on at the age of 9 and patient F6P17 had been operated on at the age of 58. While the levels of ferritin were increased, the levels also differed between patients. Table 1 shows the patients’ cataract states, whether they were operated on and at what age they were operated on, information about ferritin levels, and additional findings. Ferritin levels of siblings who were under risk were checked. In addition, the patients’ total blood count and biochemical parameters are listed in Table 2. 

**Figure 1:**
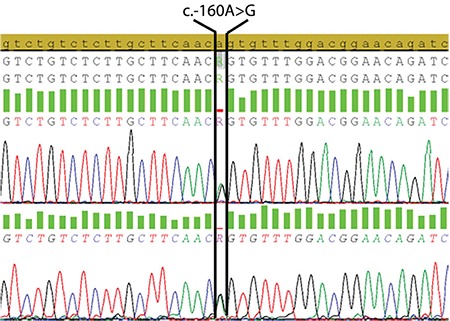
All patients had heterozygous c.-160A>G mutation in the *FTL* gene.

In the Sanger sequencing analysis conducted, all of the patients were found to have c.-160A>G heterozygous mutation in the *FTL* gene (Figure 1). This mutation is a genetic change reported in the literature previously [8].

## Discussion

Ferritin is an iron-binding protein-storing iron for vital cellular activities and it is the primary intracellular iron-storing protein in the body. The protein’s iron-free form is called apoferritin, while the iron-containing form is called holoferritin. Each apoferritin consists of 24 subunits containing an H-subunit and L-subunit.

HFCS is an autosomal dominant inherited rare genetic disease. It is characterized by early-onset cataract formation due to L-ferritin accumulation in the lens. Mutations in the 5’ untranslated region of the *FTL* gene located in 19q13.1 cause this disease [9,10]. The binding of iron-responsive elements (IREs) with iron-regulated cytoplasmic protein (IRP) forms a complex enabling the inhibition of L-ferritin levels in the 5’ untranslated region of the *FTL* gene. Mutations in the *FTL* gene disrupt the IREs binding with IRPs, shifting the iron-mediated downregulations of *FTL* translations. So far, 37 mutations have been defined in the *FTL* gene [5]. Frequent mutations are c.-168G>C/T/A, c.-161C>G/T/A, and c.-160A>G [9]. Small deletions have also been defined [11]. In our study, we found c.-160A>G mutation in 17 patients from 6 families assessed from the provinces of Kayseri and Nevşehir in Turkey. All of our patients had the same mutation. The families were not relatives. This mutation has been reported previously [2,8]. Tuysuz et al. [7] from Turkey also reported +32G>T change in 3 individuals from a family. In addition to having early-onset cataracts, these three patients had ferritin levels between 659 and 2000 ng/mL.

In HFCS, ferritin accumulates in all cells in the body. However, it causes cataracts by becoming toxic through forming L-ferritin crystal deposit formations only in the lens. The cataract is generally early-onset, bilateral, and progressive. HFCS penetration may not be complete and patients may have only hyperferritinemia. It has been claimed that the intensity of penetrance and cataract is associated with the location of the mutation [10,12]. In our study, cataracts had not developed in patients F3P7, F3P9, and F5P12, who were relatively young. However, all of the patients had hyperferritinemia. In addition, although the same mutation was found in all our patients, the differences in cataract development age and changes in ferritin levels bring to mind that factors besides the defined mutation can contribute to the disease.

One of the striking findings in this study was that the youngest patient was 1 year old and the oldest one was 72 years old. Patient F5P14, who was 72 years old, underwent several tests, including a liver biopsy. However, the patient was diagnosed with *FTL* gene analysis in our department. Early diagnosis can save time for planning the patient’s cataract surgery and it can prevent unnecessary tests.

HFCS prevalence has been shown to be around 1/200,000 in Australia [6]. All of our patients were from the provinces of Kayseri and Nevşehir in Turkey. The total population of these two provinces is about 1,700,000. We identified 17 patients from 6 families. This shows that the minimum prevalence is about 1/100,000. However, since ferritin levels were not measured routinely in cataract cases and since this study depended on hospital data, the prevalence of this disease could probably be higher.

### Study Limitations

The study has some limitations. The number of patients in the study is limited. Further studies with more patients are needed to explain why they have different ferritin levels and the age of onset of cataract.

## Conclusion

In the presence of hyperferritinemia, patients should be assessed by a hematologist and be referred to an ophthalmologist in terms of cataracts. It should be remembered that this disease is autosomal dominantly inherited and family screening should be performed. This study is the first comprehensive research article on the analysis of HFCS and FTL gene molecular analysis from Turkey. In the Turkish population, the prevalence of HFCS is about 1/100,000 and the commonly observed mutation is c.-160A>G mutation.

## Figures and Tables

**Table 1:**
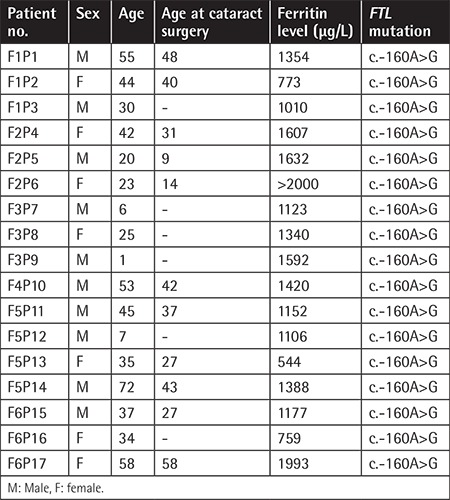
Patients’ cataract states, whether they were operated on and at what age they were operated on, information about ferritin levels, and additional findings.

**Table 2:**
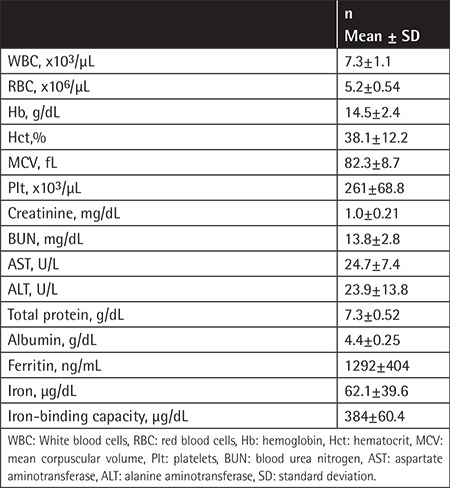
The patients’ total blood counts and biochemical parameters.

## References

[1] Hansen L, Mikkelsen A, Nürnberg P, Nürnberg G, Anjum I, Eiberg H, Rosenberg T (2009). Comprehensive mutational screening in a cohort of Danish families with hereditary congenital cataract. Invest Ophthalmol Vis Sci.

[2] Girelli D, Corrocher R, Bisceglia L, Olivieri O, De Franceschi L, Zelante L, Gasparini P (1995). Molecular basis for the recently described hereditary hyperferritinemia-cataract syndrome: a mutation in the iron-responsive element of ferritin L-subunit gene (the “Verona mutation”). Blood.

[3] Bonneau D, Winter-Fuseau I, Loiseau MN, Amati P, Berthier M, Oriot D, Beaumont C (1995). Bilateral cataract and high serum ferritin: a new dominant genetic disorder?. J Med Genet.

[4] Beaumont C, Leneuve P, Devaux I, Scoazec JY, Berthier M, Loiseau MN, Grandchamp B, Bonneau D (1995). Mutation in the iron responsive element of the L ferritin mRNA in a family with dominant hyperferritinaemia and cataract. Nat Genet.

[5] Luscieti S, Tolle G, Aranda J, Campos CB, Risse F, Moran E, Muckenthaler MU, Sanchez M (2013). Novel mutations in the ferritin-L iron-responsive element that only mildly impair IRP binding cause hereditary hyperferritinaemia cataract syndrome. Orphanet J Rare Dis.

[6] Craig JE, Clark JB, McLeod JL, Kirkland MA, Grant G, Elder JE, Toohey MG, Kowal L, Savoia HF, Chen C, Roberts S, Wirth MG, Mackey DA (2003). Hereditary hyperferritinemia-cataract syndrome: prevalence, lens morphology, spectrum of mutations, and clinical presentations. Arch Ophthalmol.

[7] Tuysuz G, Ozdemir N, Sonmez E, Kannengiesser C, Celkan T (2013). A new family with hereditary hyperferritinemia cataract syndrome. Genet Couns.

[8] Cicilano M, Zecchina G, Roetto A, Bosio S, Infelise V, Stefani S, Mazza U, Camaschella C (1999). Recurrent mutations in the iron regulatory element of L-ferritin in hereditary hyperferritinemia-cataract syndrome. Haematologica.

[9] Alvarez-Coca-Gonzalez J, Moreno-Carralero MI, Martinez-Perez J, Mendez M, Garcia-Ros M, Moran-Jimenez MJ (2010). The hereditary hyperferritinemiacataract syndrome: a family study. Eur J Pediatr.

[10] Bertola F, Veneri D, Bosio S, Battaglia P, Disperati A, Schiavon R (2004). Hyperferritinaemia without iron overload: pathogenic and therapeutic implications. Curr Drug Targets Immune Endocr Metabol Disord.

[11] Garber I, Pudek M (2014). A novel deletion in the iron-response element of the L-ferritin gene, causing hyperferritinaemia cataract syndrome. Ann Clin Biochem.

[12] Cazzola M, Bergamaschi G, Tonon L, Arbustini E, Grasso M, Vercesi E, Barosi G, Bianchi PE, Cairo G, Arosio P (1997). Hereditary hyperferritinemia-cataract syndrome: relationship between phenotypes and specific mutations in the iron-responsive element of ferritin light-chain mRNA. Blood.

